# Renal Protective Effect of DPP-4 Inhibitors in Type 2 Diabetes Mellitus Patients: A Cohort Study

**DOI:** 10.1155/2016/1423191

**Published:** 2016-12-29

**Authors:** Young-Gun Kim, JungHyun Byun, Dukyong Yoon, Ja Young Jeon, Seung Jin Han, Dae Jung Kim, Kwan-Woo Lee, Rae Woong Park, Hae Jin Kim

**Affiliations:** ^1^Department of Biomedical Informatics, Ajou University School of Medicine, Suwon, Republic of Korea; ^2^Department of Endocrinology and Metabolism, Ajou University School of Medicine, Suwon, Republic of Korea; ^3^Department of Biomedical Sciences, Ajou University Graduate School of Medicine, Suwon, Republic of Korea

## Abstract

*Aims*. Dipeptidyl-peptidase IV inhibitors (DPP-4i) are among the most popular oral antidiabetic agents. However, the effects of DPP-4i on diabetic nephropathy are not well-established. The aim of this study was to determine the renoprotective effects of DPP-4i, using albuminuria and glomerular filtration rate (GFR) as indicators, in type 2 diabetes mellitus (T2DM) patients.* Methods*. This retrospective observational cohort study used the clinical database of a tertiary hospital. The changes of urine albumin/creatinine ratio (UACR), estimated GFR (eGFR), and metabolic parameters after treatment were compared with the changes of those parameters before treatment using paired Student's *t*-test.* Results*. The mean UACR in the entire study population decreased to approximately 45 mg/g 1 year after DPP-4i treatment, while it was increased approximately 39 mg/g 1 year before DPP-4i treatment (*p* < 0.05). Patients with macroalbuminuria showed a significant reduction in albumin levels after DPP-4i treatment (*p* < 0.05); however, patients with microalbuminuria and normoalbuminuria did not show improvements in albuminuria levels after treatment. Although eGFR was not changed 1 year after DPP-4i treatment, reductions in eGFR were slowed in patients with microalbuminuria and reversed in the macroalbuminuria or normoalbuminuria groups, 4 years after treatment.* Conclusions*. Administration of DPP-4i reduces urine albumin excretion and mitigates reduction of eGFR in T2DM patients.

## 1. Introduction

Diabetic nephropathy is one of the most important complications of diabetes, being strongly associated with increased overall mortality, cardiovascular mortality, cardiovascular events, and end-stage renal disease [1, 2]. The major and earliest clinical manifestation of diabetic nephropathy is albuminuria. Albuminuria is a principal marker of kidney damage, which is caused by glomerular leakage. Generally, albuminuria is used as a marker of diabetic nephropathy; many studies have suggested that albuminuria is correlated with the progression of diabetic nephropathy, cardiovascular mortality, and all-cause mortality [3–6]. In addition, reduction of albuminuria with drugs is associated with renal protection [7–9]. However, some studies have suggested that albuminuria is not an appropriate therapeutic target for diabetic nephropathy [10]. Indeed, both loop and thiazide diuretics resulted in a reduction of albuminuria but did not improve renal outcome [11, 12]. For this reason, albuminuria and glomerular filtration rate (GFR) should be considered together as surrogate markers for diabetic nephropathy.

Dipeptidyl-peptidase IV inhibitors (DPP-4i) are among the most popular and effective oral antidiabetic agents. They have many advantages, including high glucose-lowering potency, low risk of hypoglycemia, no association with weight gain, and being tolerable in chronic renal failure patients. However, their efficacy for preventing diabetic complications, especially diabetic nephropathy, is not well-established. Physiologically, dipeptidyl-peptidase IV (DPP-4) acts on nephrons to exert various functions [13, 14], and some preclinical studies have suggested that DPP-4i exerts renoprotective effects [15–17]. However, clinical evidence regarding the renal protective effects of DPP-4i therapy is limited.

The majority of clinical studies investigating the renoprotective effects of DPP-4i have only focused on evaluating their albuminuria-lowering ability [18, 19]. Although several studies have been proposed on this topic, it is still unclear that DPP-4i slows the deterioration of GFR in diabetic nephropathy [20, 21]. Moreover, almost all of these studies only contained short-term follow-up data [18–22] and included only one DPP-4i drug; thus, they were unable to evaluate effects according to drug class.

The current study aimed to determine the renoprotective effects of DPP-4i, using albuminuria and GFR as indicators, in type 2 diabetes mellitus (T2DM) patients.

## 2. Methods

### 2.1. Study Design and Subjects

A retrospective observational cohort study was conducted using the clinical database of Ajou University Hospital, which is a South Korean tertiary hospital with 1,108 beds. The study protocol was approved by the Institutional Review Board of Ajou University Hospital.

The inclusion criteria for study participants were as follows: (1) aged ≥ 19 years with T2DM identified by the International Classification of Disease, Tenth Revision code E11; (2) prescribed DPP-4i from March 1, 2010, to February 28, 2014; and (3) data on urine albumin and creatinine levels at baseline, 1 year prior to starting DPP-4i, and 1 year after DPP-4i treatment initiation.

Exclusion criteria were as follows: glycosylated hemoglobin (HbA1c) ≤ 6.5% (48 mmol/mol) or >10% (86 mmol/mol); baseline estimated GFR (eGFR) ≤ 15 mL/min/1.73 m^2^; currently undergoing dialysis; body mass index (BMI) > 40 kg/m^2^; treatment with insulin; treatment with steroids for >7 days; and patients with dual blockade of the renin-angiotensin system (RAS).

A total of 414 patients were included in this cohort. The patients were divided into three groups according to their baseline urine albumin creatinine ratio (UACR): (1) a macroalbuminuria group (UACR ≥ 300 mg/g, *n* = 38), (2) a microalbuminuria group (30 mg/g ≤ UACR < 300 mg/g, *n* = 116), and (3) a normoalbuminuria group (UACR < 30 mg/g, *n* = 260). Additionally, we performed a long-term efficacy analysis to evaluate the effects of DPP-4i on the eGFR. Patients in the study cohort who had been prescribed DPP-4i continuously for more than 4 years and whose serum creatinine levels were measured at baseline, 4 years before and after the first prescription of DPP-4i, were included in the long-term efficacy analysis.

### 2.2. Data Extraction

The first prescription date of DPP-4i was defined as the index date, and the first prescribed DPP-4i was classified as the treatment drug in patients who were prescribed more than one DPP-4i. Cessation of DPP-4i therapy was designated as the date of changing to another antidiabetic drug, a drug prescription gap of more than 30 days, or the study end date (May 31, 2015). Drug adherence was measured using the proportion of days covered (PDC, the days of taking the medicine divided by a whole follow-up duration). PDC ≥ 0.80 was considered to indicate drug adherence and patients with PDC < 0.80 were removed from the analyses.

Demographic characteristics, including age and gender, were extracted from index data. Blood pressure, height, weight, diabetes mellitus (DM) duration, and baseline laboratory tests—including HbA1c, lipid profile, serum creatinine, urine creatinine, and urine albumin—were collected (i.e., the most recent values measured within 90-day range prior to the index date). Values for these parameters before and after treatment were also extracted using the same method. UACR was calculated using urine albumin and creatinine levels from an untimed spot urine collection. eGFR was measured using the Modification of Diet in Renal Disease Study Equation [23]:
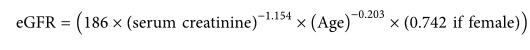
(1)


### 2.3. Statistical Analysis

All analyses were performed using R software (ver. 3.2.3; R Development Core Team, Vienna, Austria). Data are expressed as means ± standard deviation. A self-controlled design, in which comparisons are made within individuals, was used to estimate the renoprotective effect of DPP-4i. Using this method, all time-invariant confounders (e.g., sex, smoking, ethnicity, albuminuria status, other underlying diseases, and coadministrated drugs) were eliminated, and time-constant covariates (e.g., age, eGFR deterioration due to DM, and DM duration) were properly adjusted for. The paired Student's *t*-test was used to evaluate statistical differences between all parameters before and after DPP-4i treatment. Multiple linear regression analysis was performed to evaluate the effects of covariates on albuminuria reduction.

## 3. Results

### 3.1. Patients Characteristics

A total of 414 patients with T2DM satisfied the eligibility criteria of this study. The mean age of the included patients was 59.2 ± 11.5 years and the mean duration of DM was 11.0 ± 7.4 years. The mean BMI and HbA1c were 25.2 ± 3.6 kg/m^2^ and 8.6 ± 1.5%, respectively. Metformin and sulfonylurea were prescribed in 74.9% and 69.8% of patients, respectively, while 56.8% of patients were prescribed RAS inhibitors and 59.2% of patients were prescribed statins (Table 1).

### 3.2. Changes in UACR and Metabolic Parameters 1 Year prior to and 1 Year after DPP-4i Treatment

**Figure 1:**
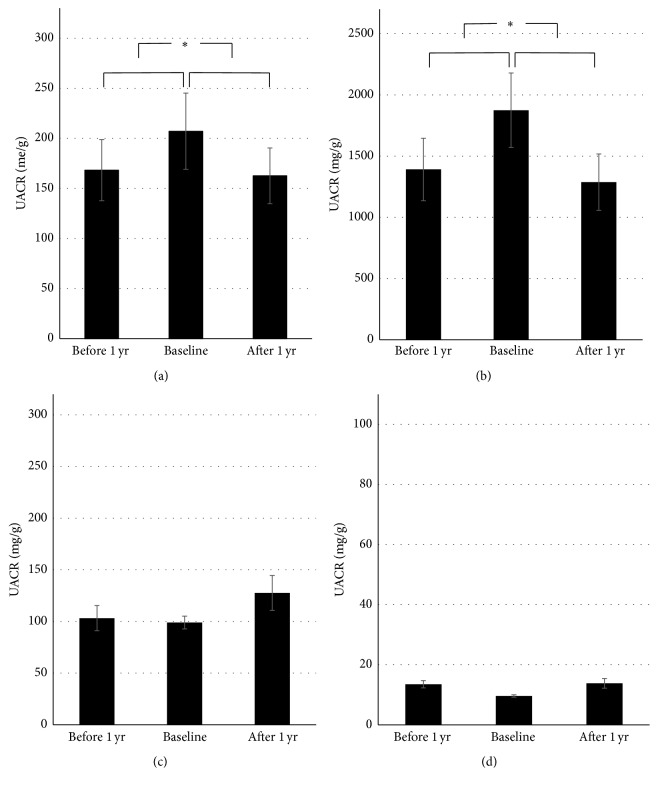
Changes in urine albumin/creatinine ratio 1 year before and 1 year after DPP-4i treatment initiation. Changes in urine albumin/creatinine ratio in all patients (a) and in patients with macroalbuminuria (b), microalbuminuria (c), and normoalbuminuria (d). (Data are presented as means with standard errors.) DPP-4i: dipeptidyl-peptidase IV inhibitor; UACR: urine albumin/creatinine ratio. ^*∗*^ *p* value < 0.05.

The mean UACR in all patients increased approximately 39 mg/g from 1 year before DPP-4i treatment to the point of DPP-4i treatment initiation, while it was decreased approximately 45 mg/g 1 year after initiation of DPP-4i treatment (*p* < 0.05). Patients with macroalbuminuria (≥300 mg/g) showed significant reductions in albuminuria (Figure 1, *p* < 0.05); however, patients with microalbuminuria and normoalbuminuria showed no significant changes.

The mean HbA1c improved from 8.6% (70 mmol/mol) to 7.8% (62 mmol/mol) (*p* < 0.01), and the mean low-density lipoprotein- (LDL-) cholesterol level decreased from 89.8 ± 39.5 mg/dL to 84.4 ± 33.1 mg/dL (*p* < 0.05). However, eGFR was not changed 1 year after DPP-4i treatment compared with 1 year before DPP-4i treatment (Table 2).

### 3.3. Estimating the Effect of Covariates on Albuminuria Reduction

To estimate the effect of covariates on albuminuria reduction, multiple linear regression analysis was performed. Although a reduction of HbA1c was shown using the paired Student's *t*-test, no significant decrease was seen on multiple linear regression analysis. Moreover, sex, age, and systolic blood pressure did not explain the changes seen in UACR on multiple linear regression analysis (Table 3).

### 3.4. Changes in eGFR 4 Years prior to and 4 Years after DPP-4i Treatment

**Figure 2:**
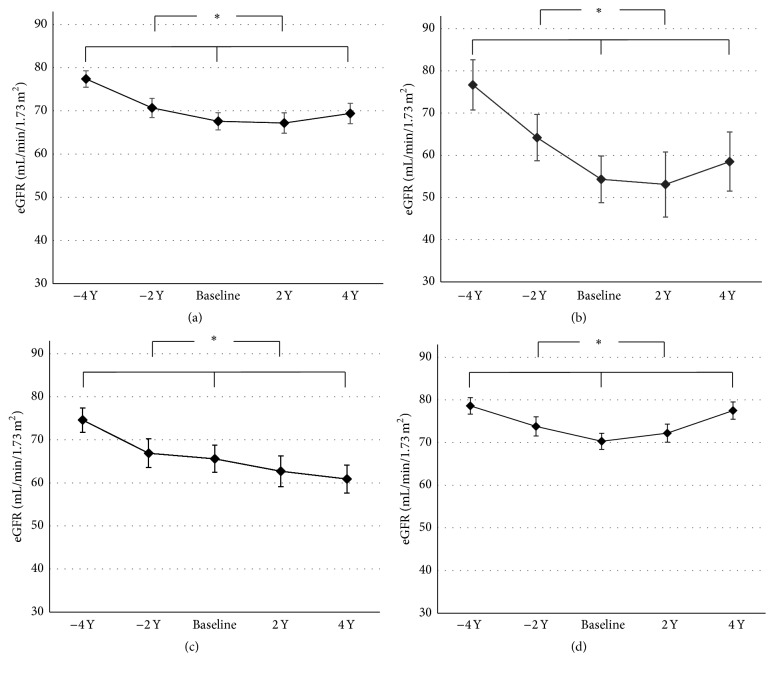
Changes in eGFR 4 years before and 4 years after DPP-4i treatment initiation. Changes in eGFR in all patients (a) and in patients with macroalbuminuria (b), microalbuminuria (c), and normoalbuminuria (d). Baseline values are the means with standard errors. DPP-4i: dipeptidyl-peptidase IV inhibitor; UACR: urine albumin/creatinine ratio; −4 Y: 4 years before DPP-4i treatment initiation; −2 Y: 2 years before DPP-4i treatment initiation; 2 Y: 2 years after DPP-4i treatment initiation; 4 Y: 4 years after DPP-4i treatment initiation. ^*∗*^ *p* value < 0.01.

To verify the long-term effects of DPP-4i on eGFR, the change in eGFR from a point of treatment to 4 years before DPP-4i treatment and 4 years after treatment was compared in patients who were prescribed DPP-4i for more than 4 years. A total of 78 patients were included in the analysis (characteristics of those patients were present in Supplementary Table  1 in Supplementary Material available online at http://dx.doi.org/10.1155/2016/1423191). The mean change in eGFR 4 years before treatment from baseline was −22.4, −9.1, and −8.3 mL/min/1.73 m^2^ in the macroalbuminuria, microalbuminuria, and normoalbuminuria groups, respectively (Figure 2). However, 4 years after DPP-4i treatment initiation, the eGFR increased in the macroalbuminuria group from 54.3 to 58.5 mL/min/1.73 m^2^ and in the normoalbuminuria group from 70.3 to 77.5 mL/min/1.73 m^2^. In each group, paired Student's *t*-test on the eGFR change from a point of treatment to 4 years before DPP-4i treatment and 4 years after treatment was statistically significant (*p* < 0.01 for all groups).

### 3.5. Subgroup Analysis for Sex, Age, Obesity, Chronic Kidney Disease Stage, and Drug Coadministration

A subgroup analysis was performed to determine which subgroup was associated with UACR changes and what factors were associated with the albuminuria-lowering effect of DPP-4i. Albuminuria significantly decreased in patients < 65 years old of both genders (*p* < 0.05). However, no significant differences in albuminuria were found when patients were divided according to their chronic kidney disease stage. Patients who were prescribed metformin, statins, and RAS inhibitors showed improvement in albuminuria (*p* < 0.05). Vildagliptin, sitagliptin, saxagliptin, and linagliptin decreased albuminuria without statistical significance (Table 4).

## 4. Discussion

This retrospective cohort study suggests that DPP-4i could reduce UACR, especially in T2DM patients with macroalbuminuria. Interestingly, DPP-4i reduced albuminuria in patients who were coadministered metformin or statins. Furthermore, DPP-4i could preserve eGFR in patients with T2DM, regardless of their baseline UACR.

Some mechanisms have been suggested to underlie the renoprotective effects of DPP-4i in previous studies. DPP-4 shows the highest expression in the kidneys among all organs and is mainly expressed in the kidney proximal tubule in healthy humans [24]. However, in DM patients, DPP-4 is also present in the renal glomerulus [25]. DPP-4 inhibition by DPP-4i was shown to reduce kidney injury in rat models of diabetes [16, 17]. One suggested mechanism underlying this effect is that DPP-4 inhibition upregulates renal cyclic adenosine monophosphate (cAMP) production by elevating circulatory stromal cell-derived factor-1a [26]. Increased cAMP has antioxidative effects and reduces reactive oxygen species, which are considered a major cause of diabetic nephropathy. Another suggested mechanism is that DPP-4i elevates active glucagon-like peptide-1, which is known to upregulate cAMP and reduce oxidative stress [27].

In our study, eGFR was increased in patients with macroalbuminuria or normoalbuminuria after taking DPP-4i (*p* < 0.01), while the eGFR reduction rate in patients with microalbuminuria was slower during the 4 years after DPP-4i initiation relative to prior to treatment initiation (*p* < 0.05). To our knowledge, this is the first study to show that DPP-4i alleviates eGFR decline. Previous studies have shown that eGFR was not improved after 3–6 months of DPP-4i treatment [20, 21]. Similarly, eGFR was not different 1 year after DPP-4i treatment but was improved after 4 years of treatment in our study. Thus, long-term DPP-4i treatment may mitigate the decline in eGFR associated with diabetic nephropathy or even improve it.

Urine albumin excretion was decreased after DPP-4i treatment in the macroalbuminuria group (*p* < 0.005) but not in the microalbuminuria and normoalbuminuria groups. This result is not consistent with previous studies, which suggested that DPP-4i had an albuminuria-lowering effect in patients with microalbuminuria [18, 21]. However, the follow-up periods of the above-mentioned studies were shorter than that of our cohort (3–6 months) and their patients were prescribed only sitagliptin. Moreover, although HbA1c was decreased after DPP-4i administration, the effect of HbA1c on lowering albuminuria was not significant in multiple linear regression. The change in albuminuria was not influenced by sex, age, DM duration, eGFR, or systolic blood pressure at baseline.

In the subgroup analysis pertaining to coadministration of other drugs, urine albumin excretion was decreased in patients who were given metformin, statins, and RAS inhibitors. The renoprotective efficacy of metformin in T2DM patients remains controversial. Some studies insist that metformin lowers urine albumin excretion and has renoprotective effects [28, 29]; however, other studies have shown that metformin did not exert these beneficial and protective effects [30, 31]. In our analysis, T2DM patients who were prescribed DPP-4i with metformin showed an improvement of albuminuria (*p* < 0.05). The combination of metformin and DPP-4i may have a synergistic renoprotective effect. However, a well-designed study with adequate power is warranted to verify this protective effect. Usually, statins disturb the uptake of plasma proteins in the glomerular tubule, which can result in albuminuria in some patients [32, 33]. In our analysis, administration of DPP-4i in patients also taking statins resulted in improvements in urine albumin excretion. Physiologically, DPP-4 and the glucagon-like peptide-1 (which is the substrate of DPP4) receptor are localized in the renal tubule. DPP-4i may prevent statin-induced proteinuria via unknown mechanisms, as well as decrease albuminuria caused by DM. However, our study was not specifically designed to evaluate any statin-induced proteinuria-lowering effect. Thus, large prospective cohort studies are needed to assess the synergistic effect of DPP-4i and statins on reduction of albuminuria.

An important strength of our study was that the cohort contained long-term treatment data. Previous studies investigating the renoprotective effects of DPP-4i used only short-term data, and they showed only albuminuria-lowering effects [18–21, 34]. Because our cohort contained patients' data 4 years prior to and 4 years following treatment initiation, we were able to demonstrate declines in eGFR being reduced and even reversed. A second strength of our study lies in the fact that we included five DPP-4i classes: sitagliptin, linagliptin, saxagliptin, vildagliptin, and gemigliptin. Although there were no significant differences between these drugs, the majority were associated with a reduction of albuminuria. It is possible that various DPP-4i classes exert different albuminuria-lowering effects in T2DM patients.

There were some limitations to our study. First, this study used a self-controlled design, as there was no control group. In this design, patient data prior to DPP-4i treatment were compared with data after DPP-4i treatment to estimate the effect of DPP-4i. Although there are some weaknesses in this design, time-invariant confounders and time-constant covariates were properly adjusted for using each patient's own data. Secondly, UACR was calculated from an untimed spot urine collection. A timed urine collection would better confirm albuminuria, as there are some diurnal variations and other conditions that affect creatinine excretion. However, timed urine collection is difficult under clinical circumstances because it is inconvenient. Unlike our retrospective study, a prospective cohort study, like MARLINA-T2D study, will be able to circumvent such disadvantages [19].

In conclusion, the present study demonstrated that DPP-4i treatment could ameliorate diabetic nephropathy, by reducing urine albumin excretion and mitigating the reduction of eGFR in T2DM patients.

## Supplementary Material

Baseline characteristics of patients included in long-term efficacy analysis of DPP-4i on eGFR.

## Figures and Tables

**Table 1:** Patient baseline characteristics (*N* = 414).

Characteristics	Results
Age (years)	59.2 ± 11.5
Sex (*n*, male/female)	224/190
Body mass index (kg/m^2^)	25.2 ± 3.6
DM duration (years)	11.0 ± 7.4
Systolic blood pressure (mmHg)	125.4 ± 17.2
Diastolic blood pressure (mmHg)	73.3 ± 10.6
HbA1c (%)	8.6 ± 1.5
LDL-cholesterol (mg/dL)	89.8 ± 39.5
HDL-cholesterol (mg/dL)	46.4 ± 12.0
Triglycerides (mg/dL)	168.0 ± 35.5
eGFR (mL/min/1.73 m^2^)	68.3 ± 17.6
Antidiabetic drugs (%)	
Metformin	74.9
Sulfonylurea	69.8
Thiazolidinedione	3.6
Alpha-glucosidase inhibitor	0.2
RAS inhibitor (%)	56.8
Statin (%)	59.2

Data are presented as means ± standard deviation or frequencies.

eGFR: estimated glomerular filtration rate; HbA1c: glycosylated hemoglobin; HDL: high density lipoprotein; LDL: low density lipoprotein; RAS: renin-angiotensin system.

**Table 2:** Changes in UACR, HbA1c, eGFR, and lipid profiles 1 year before and 1 year after DPP-4i treatment initiation.

	Changes during 1 year before treatment	Changes during 1 year after treatment	*p* value^‡^
UACR (mg/g)	40.8 ± 307.8	−44.5 ± 351.9	<0.05
HbA1c (%)	0.4 ± 1.1	−0.8 ± 1.5	<0.01
Systolic blood pressure (mmHg)	−1.2 ± 20.5	1.9 ± 20.7	0.13
Diastolic blood pressure (mmHg)	−0.3 ± 12.8	1.4 ± 12.7	0.37
LDL-cholesterol (mg/dL)	−1.2 ± 26.32	−3.5 ± 30.2	<0.05
HDL-cholesterol (mg/dL)	−0.3 ± 8.7	−0.9 ± 8.4	0.30
eGFR (mL/min/1.73 m^2^)	−0.7 ± 8.7	1.2 ± 11.3	0.69

Data are presented as means ± standard deviation.

^‡^The paired Student's *t*-test was performed to evaluate changes in each parameter from baseline to 1 year before DPP-4i treatment and 1 year after treatment initiation.

DPP-4i: dipeptidyl-peptidase IV inhibitor; eGFR: estimated glomerular filtration rate; HbA1c: glycosylated hemoglobin; HDL: high density lipoprotein; LDL: low density lipoprotein; UACR: urine albumin/creatinine ratio.

**Table 3:** Multiple linear regression analysis for predictors of change of UACR.

	*β*	*p* value
Age	−0.002	0.78
Sex (male)	0.263	0.06
Duration of diabetes	0.010	0.18
BMI	0.003	0.77
Systolic blood pressure	0.006	0.12
ΔHbA1c	0.035	0.36
ΔLDL-cholesterol	0.001	0.63
eGFR	0.001	0.98

BMI: body mass index; eGFR: estimated glomerular filtration rate; HbA1c: glycosylated hemoglobin; LDL: low-density lipoprotein.

**Table 4:** Subgroup analysis for sex, age, obesity, chronic kidney disease stage, and drug coadministration.

	*N*	UACR 1 year before treatment	Baseline UACR	UACR 1 year after treatment	UACR change during 1 year before treatment	UACR change during 1 year after treatment	*p* value^‡^
*Sex*							
Male	224	127.2 ± 401.4	168.5 ± 541.8	135.6 ± 398.1	41.3 ± 227.3	−32.9 ± 282.6	<0.05
Female	190	212.4 ± 802.2	252.5 ± 979.7	194.3 ± 714.3	40.1 ± 380.4	−58.2 ± 419.5	<0.05
*Age*							
≥65 years	133	153.2 ± 384.0	166.0 ± 551.2	151.7 ± 484.5	12.8 ± 231.3	−14.3 ± 273.4	0.66
<65 years	281	171.0 ± 715.1	226.5 ± 860.1	167.7 ± 600.9	55.5 ± 340.9	−58.8 ± 382.8	<0.05
*Obesity*							
Obese	151	162.0 ± 654.5	232.5 ± 869.5	172.3 ± 585.1	70.5 ± 437.2	−60.2 ± 433.2	0.24
Nonobese	159	206.0 ± 770.5	256.8 ± 887.4	220.6 ± 691.7	50.8 ± 267.1	−36.2 ± 348.6	0.10
*CKD*							
eGFR ≥ 90	41	131.2 ± 231.1	126.6 ± 491.6	62.2 ± 158.0	−4.6 ± 67.3	−64.4 ± 419.3	0.92
90 > eGFR ≥ 60	229	91.1 ± 496.3	104.6 ± 524.7	81.6 ± 368.7	13.5 ± 149.0	−23.0 ± 229.9	0.29
60 > eGFR ≥ 30	115	306.2 ± 652.8	338.2 ± 864.5	286.7 ± 716.5	32 ± 261.5	−51.5 ± 383.8	0.11
30 > eGFR ≥ 15	13	861.8 ± 1631.2	917.6 ± 2446.1	886.9 ± 1599.0	55.8 ± 1181.9	−30.7 ± 1074.0	0.27
*Metformin*							
Yes	310	156.0 ± 573.2	187.5 ± 678.2	137.0 ± 508.3	21.9 ± 193.7	−50.5 ± 319.8	<0.05
No	104	174.6 ± 737.5	265.3 ± 1009.5	238.7 ± 707.0	90.7 ± 495.1	−26.6 ± 435.0	0.25
*Sulfonylurea*							
Yes	289	170.4 ± 401.0	179.0 ± 560.8	153.7 ± 465.1	8.6 ± 171.4	−25.3 ± 310.1	0.46
No	125	130.9± 1039.6	271.9 ± 1122.5	182.9 ± 750.1	141 ± 537.1	−89 ± 431.5	<0.05
*Statin*							
Yes	245	201.8 ± 752.9	259.6 ± 933.4	203.5 ± 687.3	57.8 ± 382.9	−56.1 ± 398.3	<0.05
No	169	114.5 ± 352.9	131.0 ± 445.5	103.3 ± 307.2	16.5 ± 142.1	−27.7 ± 271.1	0.54
*RAS inhibitor*							
Yes	235	238.9 ± 765.7	299.3 ± 941.2	219.1 ± 645.9	60.4 ± 383.0	−80.2 ± 438.8	<0.05
No	179	73.3 ± 289.1	86.0 ± 447.5	88.4 ± 429.2	12.7 ± 140.7	2.4 ± 173.9	0.95
*DPP-4i*							
Vildagliptin	136	185.6 ± 779.5	245.1 ± 855.5	191.2 ± 618.5	59.5 ± 266.2	−53.8 ± 257.1	0.08
Sitagliptin	96	208.0 ± 181.3	209.0 ± 590.3	143.9 ± 349.2	1.0 ± 88.3	−65.1 ± 479.4	0.66
Linagliptin	77	144.8 ± 776.5	243.2 ± 1073.1	200.4 ± 724.2	98.4 ±54.3	−42.8 ± 45.3	0.30
Saxagliptin	56	233.7 ± 530.9	221.1 ± 688.9	188.8 ± 709.8	−12.6 ± 206.5	−32.3 ± 287.4	0.20
Gemigliptin	48	3.3 ± 27.1	7.2 ± 44.8	12.3 ± 63.6	3.9 ± 28.8	5.1 ± 32.0	0.60

Data are presented as means ± standard deviation.

Some patients were not included in the subgroup analyses due to missing data.

CKD: chronic kidney disease; DPP-4i: dipeptidyl-peptidase IV inhibitor; eGFR: estimated glomerular filtration rate; RAS: renin-angiotensin-system; UACR: urine albumin/creatinine ratio.
