# Supplementary material for: Renal Protective Effect of DPP-4 Inhibitors in Type 2 Diabetes Mellitus Patients: A Cohort Study
Source: J Diabetes Res. 2016 Dec 29;2016:1423191. doi: 10.1155/2016/1423191 (PMC5228170; doi:10.1155/2016/1423191)
Supplement: Supplementary file 1 — Baseline characteristics of patients included in long-term efficacy analysis of DPP-4i on eGFR. [file 1423191.f1.docx]

SUPPLEMENTARY MATERIALS

Supplementary table 1. Baseline characteristics of patients included in long-term efficacy analysis of DPP-4i on eGFR (N = 78)

| Characteristics | | | Results |
| --- | --- | --- | --- |
| Age (years) | |  | 60.1 ± 12.0 |
| Sex (n, male/female) | |  | 39/39 |
| Body mass index (kg/m^2^) | | | 22.2 ± 8.0 |
| DM duration (years) | | | 12.0 ± 6.0 |
| Systolic blood pressure (mmHg) | | | 120.7 ± 13.4 |
| Diastolic blood pressure (mmHg) | | | 69.8 ± 8.1 |
| HbA1c (%) |  | | 9.0 ± 1.3 |
| LDL-cholesterol (mg/dL) | | | 87.3 ± 23.9 |
| HDL-cholesterol (mg/dL) | | | 44.8 ± 11.9 |
| Triglycerides (mg/dL) | | | 157.5 ± 97.6 |
| eGFR (mL/min/1.73m^2^) | | | 68.0 ± 17.7 |
| Anti-diabetic drugs (%) | |  |  |
| Metformin | |  | 84.6 |
| Sulfonylurea | |  | 76.9 |
| Thiazolidinedione | |  | 2.6 |
| Alpha-glucosidase inhibitor | |  | 0.0 |
| RAS inhibitor (%) | |  | 68.0 |
| Statin (%) | |  | 66.7 |
| Data are presented as means ± standard deviation or frequencies.  DPP-4i, dipeptidyl-peptidase IV inhibitor; eGFR, estimated glomerular ﬁltration rate; HbA1c, glycosylated hemoglobin; HDL, high-density lipoprotein; LDL, low-density lipoprotein; RAS, renin-angiotensin system. | | | |
